# Biofortification in China: policy and practice

**DOI:** 10.1186/1478-4505-5-10

**Published:** 2007-09-26

**Authors:** Monica H Campos-Bowers, Brian F Wittenmyer

**Affiliations:** 1 Department of Health Management and Policy, School of Public Health, University of North Texas Health Science Center, 3500 Camp Bowie Boulevard, Fort Worth, Texas 76107, USA

## Abstract

Micronutrient deficiency undernutrition, due to insufficient levels of vitamins and minerals in the diet, remains one of the most prevalent and preventable nutritional problems in the world today. Micronutrient undernutrition is the most common form of malnutrition. Compared to the 180 million children with protein-energy malnutrition, 3.5–5 billion persons are iron-deficient, and 140–250 million persons are vitamin A-deficient. Micronutrient deficiencies diminish physical, cognitive, and reproductive development. Undernutrition is both a cause and a result of poor human health and achievement.

Middle-income nations, such as China, also suffer from micronutrient undernutrition's effects. In China's poor western provinces, despite supplementation and fortification efforts, stunting and underweight (symptoms of micronutrient undernutrition) remain common. In recent decades, nutritional adequacy, in terms of available food energy, improved immensely, as the government made food security a top priority. A potential next step for China could be to address specifically micronutrient undernutrition. The paper aims to provide a discussion of policy issues relevant to biofortification, if China were to consider the implementation of this intervention in its rural provinces.

Traditional nutritional interventions currently employ four main strategies: dietary modification, supplementation, commercial fortification, and biofortification. Biofortification, a relatively new technique, involves selectively breeding staple plant varieties to increase specific nutrient levels in plant tissues. Biofortification has the potential to provide benefits to humans, plants, and livestock; nourish nutrient-depleted soils; and help increase crop yields per acre. Biofortification methods include selective breeding, reducing levels of anti-nutrients, and increasing levels of substances that promote nutrient absorption.

If China were to implement biofortification programs, with help from government agencies and international organizations, several policy questions would need to be addressed. The paper discusses several policy questions that pertain to the relationship between biofortified and genetically modified crops, human health and safety concerns, labeling of biofortified crops for consumers, consumer rights, potential environmental impacts, intellectual property rights, seed disbursement, government investment, private-sector research, and additional agricultural and commercial regulations. Biofortification has the potential to help alleviate the suffering, death, disability, and failure to achieve full human potential that results from micronutrient undernutrition-related diseases.

## Introduction

### Magnitude of undernutrition

Poor nutritional status remains a serious problem in many regions of the world, despite many successful efforts at prevention and treatment [1]. Developing nations face severe nutritional challenges. At their roots, these challenges stem from conditions of dysfunctional food systems leading to material deprivation and deep hunger. Hungry and deprived persons suffer from a number of nutritional deficiencies and negative health conditions [1]. Compounding the challenges faced by these societies is the difficult task of implementing appropriate nutritional interventions. Compared to wealthy individuals, the world's poor disproportionately shoulder the burden of nutritional inadequacy and its health consequences.

The World Health Organization (WHO) has estimated that approximately 30% of the global population suffers from at least one form of malnutrition [2]. The prevalence of micronutrient undernutrition greatly exceeds the prevalence of protein-energy malnutrition (PEM). Compared to the global estimate of 180 million children who suffer from PEM-related wasting and stunting, an estimated 3.5–5 billion persons suffer from iron-deficiency, and 140–250 million are deficient in vitamin A [3]. Malnutrition is associated with approximately 49% of under-age-5 childhood mortality in underdeveloped nations [2].

### International development and human rights

The United Nations' Millennium Development Goals provide a set of achievable goals for reducing undernutrition and other forms of malnutrition [4]. Human rights treaties provide an ethical-philosophical foundation for nutrition-related global health projects. These agreements are potentially powerful tools that international aid organizations could use to address issues of relevance to micronutrient undernutrition, including universal access to proper nutrition, food security, and the interrelated problems of poor health. Three human rights treaties have provisions that address food security and freedom from want of the basic requirements of survival. These treaties are the Universal Declaration of Human Rights (UDHR), §25.1 [5] International Covenant on Economic, Social, and Cultural Rights (ICESCR), §11.2.a-b [6] and Convention on the Rights of the Child (CRC), §24.a-f [7]. The following Millennium Development Goals address food security, nutrition, and associated mortality and morbidity:

• "Reduce extreme poverty and hunger by half relative to 1990."

• "Reduce child mortality by two-thirds relative to 1990."

• "Improve maternal health, including reducing maternal mortality by three-quarters relative to 1990 [4]."

### Epidemiology of undernutrition

Undernutrition consists of deficiencies of protein, energy, fatty acids, and essential micronutrients and is a major dietary problem facing low-income nations. Undernutrition may be subdivided into protein-energy malnutrition (PEM) and micronutrient undernutrition. PEM consists of a continuum of dietary deficiencies, characterized by chronic protein and energy deficiencies, while micronutrient undernutrition relates to deficiencies of essential vitamins and minerals [1].

Those persons, at the highest risk for undernutrition, most often are poor and food insecure and lack many basic resources. Undernutrition has proven to be a difficult problem to solve. West, Caballero, et al have described undernutrition as the product of the "complex interaction between a diet that is chronically inadequate in protein, energy, and essential micronutrients and infection, modified by needs at certain stages of life [1]." Maternal nutritional status has important effects upon the growth, development, and health of the child, from the time of conception through birth and the first few months of life. Poor maternal and childhood nutrition negatively affects physical and cognitive development throughout the human life span. Poor childhood nutrition also increases one's chances of developing nutrition-related chronic diseases [2].

Undernutrition is both a cause and result of poorer health and the failure of an individual to achieve his or her full potential [1]. Negative health effects include reduced immune function; delayed physical, cognitive, and reproductive growth and development; and decreased ability to perform work and physical labor [3]. The severity and duration of health effects depend upon a number of factors, including specific dietary deficits, health status, environmental conditions, and stage of life [1].

Nutritional intervention strategies may be complicated by the fact that the number of calories available in the diet does not always provide an adequate reflection of the availability of essential micronutrients (vitamins and minerals). For example, calories, fats, and amino acids may be present in sufficient quantities, yet the diet may lack sufficient amounts of micronutrients. Additionally, foods often contain substances that inhibit iron nutrient absorption, such as phytates in grains that inhibit zinc absorption [1].

Micronutrient undernutrition may present as clinical and sub-clinical forms. Sub-clinical cases outnumber clinical cases, leading some persons to describe micronutrient undernutrition as a "hidden hunger [1]." One may more easily understand the magnitude of the undernutrition problem with the aid of an iceberg analogy. Clinical presentations of undernutrition are visible above the waterline. Sub-clinical cases make up the vast majority of the iceberg's mass, below the water line, that cannot be seen.

Middle-income nations, such as China, are not immune to the many effects of undernutrition, which are caused by deficiencies in energy, protein, and essential nutrients. In middle-income nations, the poor face nutritional challenges that are similar to those faced by their counterparts in less industrialized nations. In recent decades, nutritional adequacy, in terms of available food energy, has improved immensely in China, as the government made food security a top priority [8]. A potential next-step for China could be to address specifically the problem of micronutrient deficiency undernutrition. The following paper will provide a discussion of policy issues relevant to staple food crop biofortification – a method that has garnered much attention in recent years – if China were to consider the implementation of this intervention in its rural provinces.

## Discussion

### Micronutrient undernutrition remediation strategies

Currently four main strategies are used to combat micronutrient undernutrition: dietary intervention, supplementation, food fortification and biofortification. The dietary diversification strategy encourages consumers to modify their eating behaviors. By eating a wide variety of foods, consumers increase levels of needed nutrients in the diet. The strategy relies on food varieties that are already available in the population, with modifications only to a consumer's choice of foods.

Supplementation is an external nutritional intervention. Supplementation provides essential micronutrients to a target population, in the form of a vitamin pill or micronutrient-rich sprinkle. As with dietary diversification, supplementation is targeted at the individual or family level. Supplementation can be effective on a large scale, as evidenced by Indonesia's and Vietnam's successful elimination of clinical vitamin A deficiency (xeropthalmia). These successes were due in part to regular and extensive supplementation coverage. The use of medicinal approaches (supplementation) is not viable as a long-term solution. Deficiencies may reoccur in times of economic or political crisis, indicating that supplementation efforts may be subject to the effects of social instability [3].

Commercial fortification strategies are directed toward the population level, rather than the level of the individual or family. Commercial fortification involves nutritionally enriching food products by adding droplets of the micronutrients (such as iron or folic acid) directly to cereals (wheat, rice, and maize), during the first stage of milling. Considering the high level of cereal consumption globally, the use of commercial fortification has great potential as a strategy to reduce micronutrient malnutrition. For example, half of the world's land is used to cultivate wheat, rice, and maize, at the present time. It is estimated that by 2020, more than 95% of the world's rice, 66% of the world's wheat, and almost 60% of the world's maize will be cultivated within developing countries [3]. Commercial fortification has been used effectively to raise micronutrient levels in large populations. However, in developing countries in Africa and parts of Asia, where the food industry is in a rudimentary stage of development and there are few structures to ensure the quality of fortified products, commercial fortification has not been as effective as it has been in other regions. In areas where micronutrient deficiencies are common, central processing and strict quality control are often inadequate [3].

Biofortification is a strategy that uses plant breeding techniques to produce staple food crops with higher micronutrient levels [9]. These modified crops would have the ability to accumulate greater than normal amounts of vitamins and minerals and incorporate these nutrients into their edible portions. Unlike commercial fortification, biofortification does not rely on food processing or the milling process to incorporate micronutrients into the diet.

### Potential biofortification benefits and drawbacks

Biofortification can provide benefits for both human and plant nutrition. Biofortified plants have the potential to nourish nutrient-depleted soils; help increase crop yields per acre; and provide nutritional benefits to plants, humans, and livestock. The main underlying assumption for this strategy holds that there can be an increase in nutrient accumulation to plants and, subsequently, to humans, without changing consumption patterns of traditional crop staples. The main methods for biofortification include: increasing the mineral and vitamin content in food plants, via conventional selective breeding techniques; reducing levels of anti-nutrients in food staples that inhibit the absorption and bioavailability of nutrients; and increasing levels of compounds that promote the bioavailability of nutrients. Current research studies have used conventional breeding techniques to increase vitamin and mineral densities in varieties of maize, wheat, beans, cassava, and rice [10]. For a more extensive list of the potential benefits of biofortification, see Table 1.

**Table 1:** Benefits of Biofortification

Sector	Benefits
**Agricultural (crop)^a, b, c, d, e^**	1. Improved root penetration (deeper roots); increased uptake of nutrients; benefits for trace metal-deficient soils. 2. Greater Resource Efficiency: greater drought resistance, requiring less irrigation; requires less chemical fertilizer. 3. Feasibility of breeding for uptake of specific nutrients. 4. Improved disease resistance. 5. Higher yields.
**Economic ^a, b, c, f^**	1. Cost-effective (one time investment). 2. Targets rural population; provides nutrient dense foods to rural areas, where fortified foods from urban areas are not accessible. 3. Complementary to standard interventions (supplementation and fortification).
**Population^a^**	1. Feasibile (no changes choice of staple crops). 2. No behavior changes required.
**Program^a^**	Sustainable (seeds are self-fortifying).
**Sources**	a. [9] b. [10] c. [19] d. [20] e. [21] f. [3]

It is not clear if biofortified plants pose risks to the health of humans and livestock. One issue involves potential effects on the plants, themselves. One may ask the question, "Could increased levels of certain nutrients negatively affect a plant's lifecycle and generational cycle?" There is an intricate interdependence between the physiological processes of a plant that control the production, transportation, and storage of vitamins and minerals. It may be possible that the introduction of increased levels of vitamins and minerals into these pathways could have detrimental consequences to the plant [11].

One shortcoming of biofortification is its inability to provide as high mineral and/or vitamin content compared to supplementation or fortification strategies alone. However, biofortification strategies may help reduce current cost expenditures attributed to these more expensive and short-lived programs by reducing numbers of persons needing intervention. Comprehensive strategy involving multiple interventions must be adapted to specific countries and regions thus supplementation, fortification, dietary diversification and/or disease reduction programs may be helpful for biofortification to be most effective. [3,9].

### China and biotechnology

#### Agricultural biotechnology

Outside of North America, China is the current leader in plant biotechnology research. The Chinese government considers biotechnology to be the country's future and a key part of its food security policy. China increasingly views protecting this industry from foreign competition and control as an important priority. The Chinese leadership appears to be eager to maintain stability within the domestic agricultural industry, and regulations on the importation and distribution of biotechnology goods in the agricultural sector may reflect this keenness. A focus of Chinese research into agricultural biotechnology has been to find means to increase agricultural production at lower costs to farmers. The Chinese have made efforts to create crops with resistance to disease, drought, and insect pests. Such improvements could enable China to expand its supply of arable land.

After China joined the World Trade Organization (WTO), it came under pressure to allow the importation of low-cost agricultural products. The Chinese leadership is eager to ensure stability in its agricultural sector and prevent unrest among its very large number of rural unemployed. China wishes not to become dependant on foreign supplies for seeds and agricultural products, yet it desires to attract foreign technology and investment. However, even in its relationships with foreign suppliers, it wishes to ensure full government control over product development and to keep out direct competition to its biotechnology industry for as long as possible [12].

#### China and biofortification

China has enacted agricultural and economic reforms and programs to reduce poverty [13]. Areas of concern, which could hamper the achievement of this goal, are an increasing population and decreasing supply of arable land [14]. Globally, at least 50% of the arable land that has been allocated for crop production is low in the availability of one or more essential micronutrients [13]. Approximately 20% of the overall rural population is vitamin A-deficient, and approximately 40% of rural, childbearing-aged women suffer from iron deficiency anemia (IDA) [15,16]. Currently, stunting and underweight, symptoms of micronutrient undernutrition, remain high in China's poorer provinces, despite supplementation and fortification efforts. Increasing crop production may not be enough to relieve these deficiencies.

Current studies are underway, with the International Food and Policy Research Institute (IFPRI), to analyze the impact of change on the economy and agricultural sector. Topics of interest include: the impact of China's entrance into the World Trade Organization (WTO) on rural farmers in the northwest and southwest regions, measurements of current agricultural infrastructure and support (including pricing and financial systems), and an examination of soil conditions and their impact on agricultural production. A program of interest is the HarvestPlus Program of the Consultative Group on International Agricultural Research (CGIAR). This program's research is geared toward rural farm areas and has examined the impact of plant breeding on enhancements of micronutrient levels in Southeast Asian diets. IFPRI's research has revealed that important parts of successful agricultural efforts are government investment, educational assistance, and technical expertise. If this program were to be introduced into China, the most cost-effective ways to reduce rural poverty and increase the growth of the Chinese agricultural sector may be to increase public-sector investments in education, agricultural research and development, and road-building programs. Governments can encourage private-sector investment in the agricultural sector by improving individual farmer-level access to financial capital, equipment and infrastructure, agricultural education, and technical expertise [13].

#### China food policy

The Chinese government has a number of ministries that have the power to regulate food safety. The Ministry of Health is the standard regulatory agency in the food and agriculture industry. China's 1995 food safety legislation allows the Ministry of Health to oversee food safety by regulating food labeling, quality, safety, and packaging [8]. In conjunction with the Ministry of Agriculture, the Ministry of Health sets policies for the agricultural use of chemical pesticides and labeling of organic foods, even those foods that may have been contaminated by pesticide residues [8].

Before 2000, China's regulatory policies did not specifically address issues that were related to transgenic foods. Examples of these issues were consumer rights and the safety of transgenic foods [8]. Also, there was no intact process that detailed the manner in which consumers were to be informed that certain foods had been transgenic. To address the absence of clear guidelines, China created a separate set of regulations to cover transgenic foods. In May 2001, China's State Council implemented the Regulation Concerning the Biotech Safety Management of Agricultural Gene Alteration. This regulation instructed that transgenic technologies were to be assigned a human health risk level, and that assessments shall be made of potentially harmful effects that these plants could have on humans and other organisms. The regulation required labeling of products made with transgenic crops, in order to increase consumer awareness [8]. Several government ministries support labeling transgenic food products: the Ministry of Agriculture (marketing and information), Ministry of Health, Ministry of Science and Technology, Customs Office (import and export inspection), Ministry of Light Industry (food processing regulation), and the Bureau of Domestic Commerce [17].

The Regulation Concerning the Biotech Safety Management of Agricultural Gene Alteration does not address several food safety issues, such as issues related to allergies and bioavailability. In addition, the regulation does not require higher standards for screening transgenic products, compared to standard food-screening protocols. The regulation directs the Committee on Safety to require laboratory screening of transgenic food crops, prior to commercial release. The screening process includes a 30-day period of standard toxicity testing in one of the Ministry of Health's laboratories [8].

#### Intellectual property rights

Many biotechnology advocates look toward China as a country that provides hope for the use of transgenic food technologies in developing nations. Up to the present time, China has demonstrated greater permissiveness and acceptance of transgenic crops than have many other nations. In many other areas – notably Japan and Europe – consumers, the general public, and environmental groups have expressed limited enthusiasm and a great many concerns about the safety of these technologies [8]. The United States has shown greater acceptance of transgenic food technologies, though many American environmental and consumer rights groups have expressed concern.

China has shown greater speed in the development and commercial introduction of transgenic plant varieties, though, as of 2001, it had been unwilling to allow the sale of transgenic plant foods directly to consumers. Furthermore, the intellectual property rights (IPR) climate in China has tempered, to a certain extent, the willingness of foreign multinational corporations and domestic, privately held companies to develop transgenic plant technologies. The Chinese government has done much in recent years to first adopt and then abide by the minimal standards for international IPR regulation. With respect to the ability to patent transgenic plant technologies, the Chinese IPR legal climate has shown a greater resemblance to that found in Europe than to the American patent system. The Chinese government has viewed plant biotechnology as one method of maintaining domestic food self-sufficiency and security and has placed great confidence in the technical abilities of its researchers [8]. Biotechnology may be one method by which the Chinese may increase micronutrient levels in staple grains, such as wheat and rice, in order to reduce the prevalence and effects of micronutrient deficiencies among its population.

The Chinese government has made significant progress in developing its IPR framework, though it has yet to fully embrace IPR policies that would entice large-scale foreign investment or the introduction of transgenic plant varieties into China. Despite progress in this direction, sizeable obstacles remain, due to lax enforcement of IPR regulations and official mistrust in some government quarters. Chinese patent laws are similar to European regulations, as plant and animal varieties and products that violate 'public order or morality' cannot be patented. Despite the weaknesses of Chinese IPR protections, the size of its markets has enticed some foreign firms to introduce transgenic varieties. Often these varieties have been limited to hybrids, rather than freely pollinated varieties [8].

Bt cotton is an example of a transgenic plant variety. The case of Bt cotton is illustrative of China's relative eagerness to exploit the promise of transgenic technologies and the difficulties experienced by some foreign multinational corporations in China. By 1997, China had quickly approved and introduced Bt cotton varieties, developed independently by Monsanto and Chinese public-sector firms, to the marketplace. Conversely, by 2001, India had not allowed the introduction of Monsanto's crop. Chinese farmers were quick to embrace Bt cotton for its potential to provide savings on labor and pesticide application costs. Despite its popularity, Monsanto made relatively little money from its plant, as there was widespread piracy, saving, and distribution of seeds by Chinese farmers and seed suppliers. The Chinese government's hesitation to introduce stronger IPR protections to foreign and domestic firms may be influenced, in part, by the government's support of research by Chinese scientists. Despite the weaknesses of Chinese IPR protections, the sheer size of the Chinese market and the profits to be made may entice further investment by foreign and privately held Chinese biotechnology firms [8].

## Conclusion

### Food safety policy: biofortification policy implications

National policy interventions would be needed if China were to effectively implement a biofortification program in its rural areas. One issue to address regards the commercialization of biofortified crops and products: should biofortified crops be labeled as GM crops and be subject to the same or similar regulations? Under the Regulation Concerning the Biotech Safety Management of Agricultural Gene Alteration, GM crops are to be tested for toxicity prior to commercialization. In the biofortificaton process, there are no extensive genetic alterations of the plant; thus, these organisms should not be considered "GM [8]." However, because levels of certain micronutrients are higher in biofortified crops, an amendment is needed to the Regulation Concerning the Biotech Safety Management of Agricultural Gene Alteration. This amendment should address precautions that guard against excessive vitamin levels and promote standard vitamin maxima.

A second issue involves the labeling policies that are specific to Chinese biofortified crops and agricultural products: should Chinese biofortified crops and agricultural products be labeled as such if commercialized? Currently, the Regulation Concerning the Biotech Safety Management of Agricultural Gene Alteration requires the labeling of genetically modified foods [8]. Labeling should be considered, because of the increased vitamin levels in biofortified crops.

### Intellectual property rights: biofortification policy implications

The issue of intellectual property rights requires addressing, both before and after the implementation of biofortification programs. If biofortification were to be introduced into the rural areas of China, an important issue would be the best way in which to protect IPRs. In 1998, the State Intellectual Property of Office (SIPO) in China was established to enforce IPR regulations. Today, SIPO is responsible for granting patents (national office), registering semiconductor layout designs (national office), and enforcing patents (local SIPO offices), as well as coordinating domestic and foreign-related IPR issues that involve copyrights, trademarks, and patents. Currently, multiple agencies have jurisdiction over IPR regulation and enforcement [18]. Patent registration and enforcement should be reformed, to allow for coordination and enforcement across agencies and jurisdictions. Once this infrastructure has been established, the Chinese government should have the ability to improve IPR protections for biofortified crops. Caps on seed prices and the rights of Chinese farmers to sell seeds locally, nationally, and internationally should be considered.

### Additional policy needs

Another critical issue involves the manner in which China's trade policies address biofortification issues. With respect to biofortification, China would need to establish policies regarding limits on the importation of biofortified seeds and to balance its responsibilities, as a member of the WTO, with its need to protect its domestic agricultural industry.

More research is warranted on the impact of biofortification efforts and their effects on the environment. Research is needed into the drafting and implementation of agricultural policies and regulations, in order to improve and protect biofortified crops in China. A number of important measures could improve soils and protect crops. In order to decrease the incidence of cross-pollination, guidelines should be established which address the appropriate distances between separate biofortified crop fields, between biofortified and non-biofortified fields, and between biofortified crop fields and stands of wild varieties of the same species. Crop rotation should be encouraged as a standard practice among farmers of biofortified crops, in order to sustain and distribute nutrients evenly in the soil. In addition, biodiversity should be encouraged during fallow seasons. Biodiversity, in this context, refers to the cultivation and plowing-under of grasses or other ground cover to improve field fertility. A prudent strategy, to preserve locally used and traditional (pre-biofortified) crops in the region, would be the establishment and maintenance of a centralized germplasm bank.

Biofortification efforts worldwide have the potential to be effective at decreasing the prevalence of micronutrient deficiency-related diseases and improving the public's over-all health. To enhance the effectiveness of biofortification strategies, governments should recognize the benefits and consider providing structure through nutrition and agricultural policies. To date, China has made progress in increasing agricultural productivity, in order to obtain and establish food security. Despite this progress, micronutrient deficiency undernutrition remains an important issue in rural areas. Biofortification offers a promising opportunity to reduce micronutrient undernutrition and improve the health of China's rural poor. To be successful in this endeavor, China should begin now to prepare its infrastructure for such an intervention.

## Competing interests

The author(s) declare that they have no competing interests.

## Authors' contributions

MHC conceived of the study, participated in the design and co-ordination of the study, helped to perform the literature search, and helped to draft and prepare the manuscript. BFW participated in the design and co-ordination of the study, helped to perform the literature search, and helped to draft and prepare the manuscript. Both authors read and approved the final manuscript.
